# Use of explicit priming to phenotype absolute pitch ability

**DOI:** 10.1371/journal.pone.0273828

**Published:** 2022-09-14

**Authors:** Jane E. Bairnsfather, Margaret S. Osborne, Catherine Martin, Miriam A. Mosing, Sarah J. Wilson

**Affiliations:** 1 Melbourne School of Psychological Sciences, The University of Melbourne, Melbourne, Victoria, Australia; 2 Melbourne Conservatorium of Music, The University of Melbourne, Melbourne, Victoria, Australia; 3 Department of Neuroscience, Karolinska Institutet, Stockholm, Sweden; 4 Behaviour Genetics Unit, Max Planck Institute for Empirical Aesthetics, Frankfurt am Main, Germany; University of Toronto, CANADA

## Abstract

Musicians with absolute pitch (AP) can name the pitch of a musical note in isolation. Expression of this unusual ability is thought to be influenced by heritability, early music training and current practice. However, our understanding of factors shaping its expression is hampered by testing and scoring methods that treat AP as dichotomous. These fail to capture the observed variability in pitch-naming accuracy among reported AP possessors. The aim of this study was to trial a novel explicit priming paradigm to explore phenotypic variability of AP. Thirty-five musically experienced individuals (*M*_age_ = 29 years, range 18–68; 14 males) with varying AP ability completed a standard AP task and the explicit priming AP task. Results showed: 1) phenotypic variability of AP ability, including high-accuracy AP, heterogeneous intermediate performers, and chance-level performers; 2) intermediate performance profiles that were either reliant on or independent of relative pitch strategies, as identified by the priming task; and 3) the emergence of a bimodal distribution of AP performance when adopting scoring criteria that assign credit to semitone errors. These findings show the importance of methods in studying behavioural traits, and are a key step towards identifying AP phenotypes. Replication of our results in larger samples will further establish the usefulness of this priming paradigm in AP research.

## Introduction

The ability to recognise and label the pitch of a musical note is an important component of musicianship. For most musicians, this is achieved using contextual cues, by identifying the relative distance between the pitches of notes in chords and melodies (relative pitch; RP). Some, however, can additionally identify and label musical pitches in isolation, without using an external reference pitch. This skill is known as perfect or absolute pitch (AP), and has been linked to differences in brain structure and function (for a recent review, see [1–6]). Although AP is commonly conceptualised as a binary trait [7–11], variability in pitch-naming ability has been widely reported [12–22], prompting speculation that AP skill lies on a continuum rather than constituting a distinct categorical trait.

A definitional framework for AP has previously been proposed by Levitin [23]. In this model, AP comprises the separate abilities of *pitch memory*, or long-term stable memories for musical pitches, and *pitch labelling*, the association between the pitch and a specific verbal label. Good pitch memory appears to be reasonably common, even among those without musical training. For example, 40% of a general population sample had good pitch memory for familiar songs, singing them at approximately the correct pitch from memory [23]. Furthermore, adults and children as young as four can distinguish the canonical pitch of familiar melodies from pitch-shifted versions [24, 25]. This suggests that a latent form of AP may be present in the general population independent of musical training [26–28]. Indeed, pitch processing using absolute rather than relative pitch cues is accessible to infants [29, 30], demonstrating that the pitch memory skills required for AP are not unique to musicians with AP.

While good pitch memory may be present even among non-musicians, in musicians this can sometimes be accompanied by a degree of pitch labelling. These incomplete AP profiles are variously referred to as partial or quasi-AP (QAP), and include those who score above chance on pitch naming tasks, but below *a priori* accuracy ‘thresholds’ for AP. These musicians are thought to have incomplete internal pitch templates consisting of some but not all chroma (pitches referred to by note names, such as F sharp or D) [20]. Moreover, “partial” or “white-key note AP”, described by Miyazaki [31], refers to those musicians who have AP for the chroma corresponding to the white keys of the piano only, a pronounced form of an overall white-key advantage seen in both accuracy and response time even among high-accuracy AP possessors [32–35].

Partial expressions of AP are not only seen at the behavioural level, but are also evident in neuroimaging studies. The exaggerated leftward asymmetry of the planum temporale in AP musicians [36–38] is absent in those with QAP, with these musicians showing a greater degree of planum temporale symmetry than musicians either with or without AP [20]. QAP musicians also recruit a broader frontotemporal network than AP musicians when making pitch judgements, suggesting an enhanced role for pitch working memory processes to support incomplete pitch templates and thus, increased RP judgements [20]. Since RP judgements involve the use of a known reference tone to infer the identity of presented notes, this comparison process would necessarily involve working memory as the reference tone needs to be ‘held in mind’. In contrast, while a lesser working memory load has been proposed for pitch identification judgements in AP musicians, the development and maintenance of the AP pitch template still likely requires auditory working memory processes [39]. A reduced or absent P300 component in event-related potential (ERP) paradigms provides evidence for this reduced role [40–44], although the effect has not been consistently reported, which may be partially attributable to differing criteria for AP group membership across studies [45, 46]. In either case, QAP musicians do not display this reduced P300 component [47], suggesting a more central role for working memory in these intermediate performers. QAP musicians may utilise working memory to keep one or more reference chroma in mind, thus combining AP and RP strategies to identify unknown pitches. Although such strategies have been reported in QAP [20], they are yet to be systematically examined. Moreover, some chroma have been described as common references for those with QAP, such as C, G, and A [20, 48, 49], while others describe a general preference for white-key notes [19]. In other words, the nature of the QAP pitch template appears to vary among individuals, highlighting the need for a comprehensive investigation into individual templates and the factors influencing their formation.

The precise combination of factors required for the expression of AP is unknown, however, there are multiple predisposing factors encompassing both environmental and genetic influences. Early onset of musical training is widely established as necessary [50–54], and growing evidence suggests a heritable component (see [55] for a review). Other influences include the type of musical pedagogy [56, 57], choice of primary instrument [50, 57], current musical practice [33, 50], and having Asian ethnicity/language background [56, 58–60]. Rather than any specific influence, Wilson et al. [50] showed that the presence of a greater number of factors was associated with a higher likelihood of possessing AP ability. Consistent with this, AP musicians tend to report more factors than QAP musicians, who in turn report more factors than non-AP musicians [50]. This apparently cumulative nature of environmental and genetic influences on pitch-naming ability is consistent with phenotypic variability of AP.

Although QAP has been documented in musicians, current methods for assessing AP have generally not focused on QAP and thus our understanding of the nature of QAP pitch templates is limited. A somewhat circular definition of AP has persisted, whereby ‘failure’ on a pitch-naming test places musicians into a non-AP category, reinforcing an AP dichotomy and neglecting examination of the mechanisms underlying variable performance on AP tests. Moreover, accuracy thresholds for AP possession vary considerably across studies, with AP musicians variously classified as those who surpass 90% [10], 85% [58], 80% [61], or 60% [62] on pitch naming tasks, or who self-report as having AP [38]. The use of differing instruments or synthesised pitches for AP task stimuli also reduces the comparability of accuracy thresholds across studies, as pitch-naming accuracy can fluctuate based on stimulus timbre and in extreme octave ranges [19, 34, 63–66]. A further complication is a difference in scoring practices between studies, with some assigning full or partial credit for semitone errors [7, 53, 58]. Assigning partial credit improves the scores of those who frequently make small errors, while having little effect on those who perform at chance level, thus stripping variability from the distribution and likely exaggerating the perception of bimodality.

These procedural and conceptual discrepancies in the AP literature, in combination with the rarity of the trait, hinder the search for the genetic contributions to AP. While AP is likely partially heritable [8, 10, 53, 56, 67] and chromosomal loci of interest [68] and a potential candidate gene [69] have been identified, we currently lack consensus on the nature of AP phenotypes. In working towards this, examining the full range of AP abilities allows phenotypic variability to be explored. In other words, examining the spectrum of AP using methods that capture intermediate performance allows more precise characterisation of AP phenotypes.

Since QAP musicians partly rely on RP strategies to label notes, one method that probes the AP spectrum is to identify those chroma that form part of a QAP musician’s pitch template versus those that can only be labelled using RP, to look for systematic effects. One technique for achieving this is to use an explicit priming paradigm. Priming studies involve the presentation of an additional (prime) stimulus prior to a target, with the intent to activate relevant semantic categories and thus increase the likelihood of a particular response to the target [70–72]. Priming has been previously used in the study of expectations around harmonic structure [73–76], however, to our knowledge it has not been used in relation to pitch naming. Since AP representations of chroma are thought to utilise semantic memory [39, 77], an explicit priming paradigm may allow those tones that form part of a QAP musician’s pitch template to be differentiated from those that require RP judgements based on the accuracy of pitch naming in response to primed stimuli. This therefore provides a novel way to access information about individual QAP pitch templates.

In particular, our novel explicit pitch priming paradigm was designed to test cognitive representations of reference chroma in individuals with partial pitch templates (QAP) since target identification following the sounding of these chroma will be superior to when an individual must first consciously recall the reference pitch. When an intermediate performer correctly identifies a chroma in a traditional pitch-naming task, it is difficult for the researcher to discern whether the participant accessed this chroma in their internal pitch template (using AP), or whether they used a reference chroma to deduce the identity of the pitch through RP methods. The priming task differentiates between these two states by determining those target chroma that can be identified regardless of the prime (and thus form part of a participant’s pitch template) versus those that are facilitated by the sounding of the prime (and thus rely on the identity of the prime). This method allows systematic quantification of the nature of pitch templates in individuals with varying AP ability, leading to the potential identification of AP phenotypes. A further benefit is the removal of arbitrary and variable *a priori* thresholds for defining AP ability.

In this proof-of-concept study for our novel priming task, we expected that intermediate-performing participants would be most likely to show differing pitch templates. By their nature, near-ceiling level (AP) and chance-level (non-AP) participants would show less variability as all prime and target chroma would be uniformly highly accurate (AP) or inaccurate (non-AP). We used the data-driven approach of latent profile analysis (LPA) to identify similarities in pitch templates across the sample that may constitute different AP phenotypes. Data were derived from both our explicit priming AP task (AP-P) and a standard AP task (AP-S) to capture the range of pitch naming performance, keeping in mind that chroma that form part of a participant’s pitch template will show similar levels of accuracy across both tasks. Using this approach we predicted that differing AP phenotypes would be reliably identified. These phenotypes are intended to be interpreted in the context of the current sample, rather than as broad generalizations regarding an AP spectrum. As a preliminary validation of these phenotypes, we then performed exploratory analyses investigating how these phenotypes relate to factors known to influence the expression of AP, such as current and previous musical training, ethnicity, and a family history of AP. Finally, to examine the influence of different scoring methods of AP, we performed LPAs for both raw pitch-naming accuracy scores and scores with semitone (ST) errors coded as correct.

## Methods

### Participants

Thirty-seven musically experienced participants were recruited from the University of Melbourne via advertisements placed on student noticeboards. Advertisements did not specifically target AP possessors so as not to exclude individuals with uncertain pitch naming ability. A small, lab-based sample was chosen over a less-controlled but larger online study, in order to develop a robust and highly reliable priming task in a carefully controlled experimental environment. A brief questionnaire was used to screen participants for a history of hearing, neurological or psychiatric disorders, resulting in the exclusion of one participant due to significant hearing impairment. One further participant withdrew before completing the tasks. Table 1 shows demographic and musical training variables for the remaining 35 participants. The study was approved by the University of Melbourne Human Research Ethics Committee and all participants provided written, informed consent.

**Table 1:** Demographic characteristics and music experience of the participants.

	Participants (*N* = 35)
Age (mean years, range)	28.9 (18–68)
Male (n, %)	14 (40.0%)
Asian ethnic background (n, %)	11 (31.4%)
First language tone language (n, %)	6 (17.1%)
Age commenced music training (mean years, range)	5.6 (3–11)
Music training (mean years, range)	11.1 (0.5–20)
‘Fixed do’ music training (n, %) ^a^	12 (34.3%)
Music theory training (mean years, range)	4.9 (0–15)
Main instrument fixed pitch (n, %)	28 (80.0%)
First instrument fixed pitch (n, %)	30 (85.71%)
Current music practice (mean hours per week, range)	8.66 (0–21)
Current music listening (mean hours per week, range)	19.74 (3–70)
Family history of AP (n, %)	4 (11.43%)

^a^ ‘Fixed do’ refers to a method of training emphasising the consistent mapping of tones to note names (as opposed to ‘moveable do’, in which pitch to label mapping varies depending on the musical key). Fixed pitch instruments are those with fixed tunings, such as piano, compared with instruments with pitch that varies based on the musician’s skill (for example, string and woodwind instruments).

### Materials and procedure

Demographic and musical background information was obtained using the *Survey of Musical Experience* [78] with supplementary questions relating to AP (see Table 1 for variables of interest). All participants were initially tested for AP using a test of pitch naming accuracy similar to others used in previous research, comprising 50 synthesised piano tones between C2 and C5 (A4 440 Hz; for further details, see [20, 50]). Chroma were presented in randomised order, and each trial consisted of a 500 ms tone followed by 2500 ms interval for verbal response. Individuals set the stimulus volume to a comfortable level. Participants responded with either the chroma name (no octave required) or “pass” for each trial, and were permitted to self-correct, with the correction taken as the final response. Verbal responses were recorded as WAV files (44.1 kHz) using a digital audio recorder with inbuilt stereo microphone and were analysed using ProTools LE 7. This AP-S task was used to classify individuals according to previously published AP research methods, and provided a reference point against which to compare performance of the novel AP-P task.

**Fig 1:**
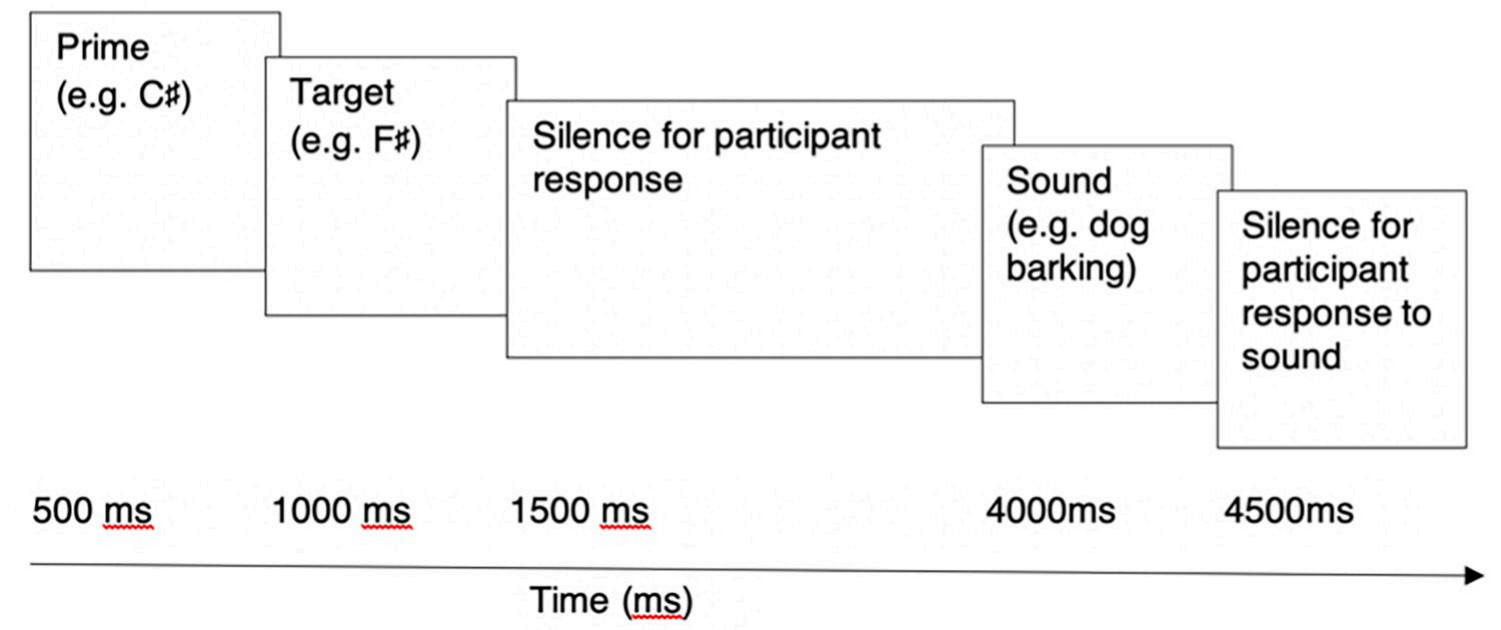
Experimental paradigm for the explicit priming AP task (AP-P).

The AP-P task (Fig 1) comprised pairs of synthesised piano tones drawn from the central pitch range C3 (130.81 Hz)–C5 (523.25 Hz) and constructed and presented using the same equipment as used for the AP-S task, as described in Wilson et al. [50]. An initial 500 ms “prime” tone was followed by a second “target” (500 ms) to be verbally labelled by participants. No feedback was provided regarding the identity of either prime or target. Following a 3500 ms response interval, an environmental sound without a clear pitch was presented for verbal identification, which served as a distracter item to limit pitch interference between trials. Sounds included vehicle noises, human sounds (e.g. coughing, laughing), animal noises, percussion instruments, tools and other sounds such as breaking glass and gunshots. Audio clips of environmental sounds were gathered from a variety of sources or recorded live, edited to 1000 ms in length and normalised to provide consistency across items.

**Fig 2:**
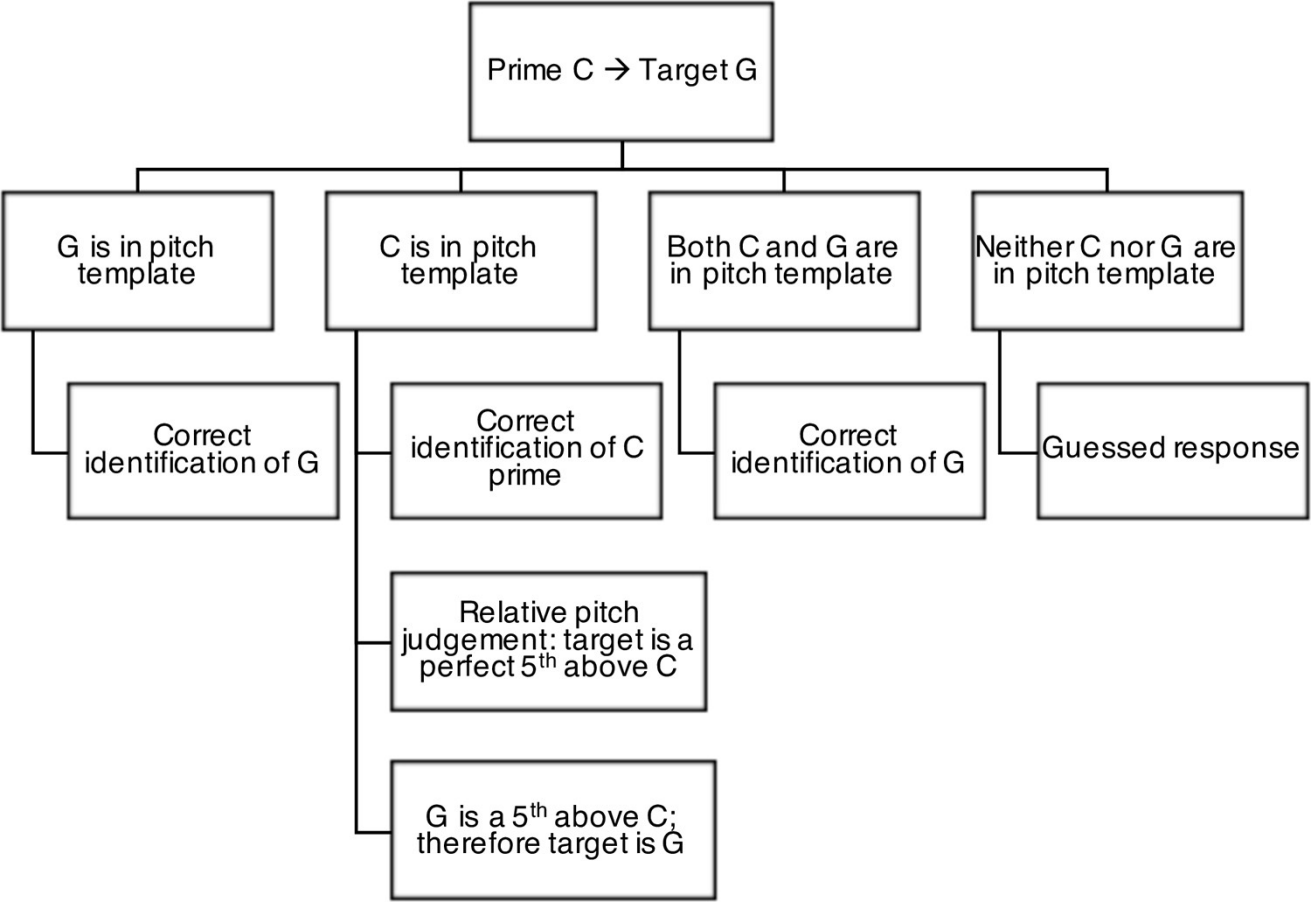
Decision tree for the explicit priming AP task (AP-P). The first line shows the presented stimuli–a C prime, followed by target G. The second line shows the hypothetical options for responding, given differing chroma in a participant’s pitch template. The third line shows the outcome from each possible scenario. Note that chance performance corresponds to 1/12, or 8.33%.

Explicit priming trials were presented in quasi-random order with each of the 12 chroma paired once with each of the 11 remaining chroma to create a prime-target pair, producing a total of 132 trials presented in two blocks of 66 to limit fatigue effects. Fig 2 shows a decision tree for possible outcomes of a single trial containing the chroma C as the prime and G as the target.

Tasks were administered to participants in an anechoic chamber via two speakers in free field set at a comfortable listening level. Verbal responses were recorded using a digital audio recorder with inbuilt stereo microphone and coded according to a range of response accuracy measures, as described in Table 2. Response times were also recorded but were not the focus of this study.

**Table 2:** Accuracy variables derived from the standard AP (AP-S) and explicit priming (AP-P) tasks.

Variable	Description
AP-S task accuracy *(ST)*	Participant’s total accuracy score for the standard AP task (maximum = 50).
AP-P task accuracy *(ST)*	Participant’s total number of accurate target identifications for the explicit priming AP task (maximum = 132).
AP-S chroma accuracy *(ST)*	AP-S task accuracy, divided into individual accuracy scores for each chroma. Scores were a maximum of 4 (or 5 for C and D♯) for each chroma.
AP-P target chroma accuracy *(ST)*	AP-P task accuracy, divided into individual accuracy scores for each target chroma (maximum = 11 per chroma).

*Note*. *(ST)* following the variable name indicates that there is both a raw and semitone (ST) version of this variable. *ST* versions refer to scores that additionally count responses either one semitone above or below the presented chroma as correct (for example, acceptable answers for the chroma G would be F♯, G, and G♯).

### Data analysis

#### Phenotype identification: Group data

To test the prediction that differing AP phenotypes would be reliably identified, latent profile analyses (LPAs) were conducted using MPlus Version 8 [79] on AP-S chroma accuracy and AP-P target chroma accuracy. These analyses determined how variability in pitch-naming ability might be optimally divided into groups, using model fit statistics to evaluate the differences between suggested numbers of groups. LPAs were conducted on both tasks to see how AP-P and AP-S phenotypes would compare. LPAs were conducted with one categorical latent variable (the grouping variable) and the twelve chroma per participant as dependent variables. Model comparisons were conducted using the Akaike’s Information Criterion (AIC) [80], the Bayesian Information Criterion (BIC) [81], and the Adjusted Bayesian Information Criterion (ABIC) [82], with smaller values representing better model fit.

Cluster analyses are common methods for identifying groups within data [83]. We chose to perform LPAs on our data in this instance as they are less reliant on subjective interpretation than cluster analysis, have formal model comparison measures, and present a more statistically robust alternative [84]. Given our small sample, our analyses are initial endeavours in examining AP data through a data-driven lens. As LPAs are generally advised to be performed with larger samples [85] we additionally performed a hierarchical cluster analysis on AP-S chroma accuracy to validate the solutions generated by our LPAs. We performed a hierarchical agglomerative cluster analysis, using the squared Euclidian distance measure and average linkage method [84, 86].

#### Phenotype identification: Individual data

To explore individual pitch templates in AP-P, we ran logistic regressions for each participant using the chroma of each trial’s prime and target to predict the likelihood of correct target identification (thus including *n* = 132 trials for each participant’s regression analysis). Chroma that emerged as significant predictors of response accuracy in the regression analyses enabled individual pitch templates to be constructed. For target chroma and prime chroma predictors, C was chosen as the statistical base group, such that the ability of chroma (as either targets or primes) to predict a correct response was compared with the predictive ability of C (as a target or prime). The selection of a base group was necessary for the construction of the logistic regressions. Three models were compared: a base model with no predictors, a model with target chroma as a predictor, and a model adding prime chroma as a predictor. This third model allowed for the examination of the use of RP strategies in pitch identification, through predicting the accuracy of target identification following specific primes.

#### Factors associated with AP ability

Based on the groupings identified by the AP-P LPAs, exploratory chi-square analyses (Fisher’s Exact Test) and a one-way analysis of variance (ANOVA) were conducted to investigate which demographic and musical experience variables differed between groups.

#### Impact of scoring methods

To analyse the effect of different scoring protocols on resultant phenotypes, we ran additional LPAs using AP-S chroma accuracy ST and AP-P chroma accuracy ST.

All analyses aside from LPAs were performed using IBM SPSS Statistics 24 with a 5% significance level used throughout.

## Results

### Phenotype identification

#### Comparison of AP-S and AP-P

**Fig 3:**
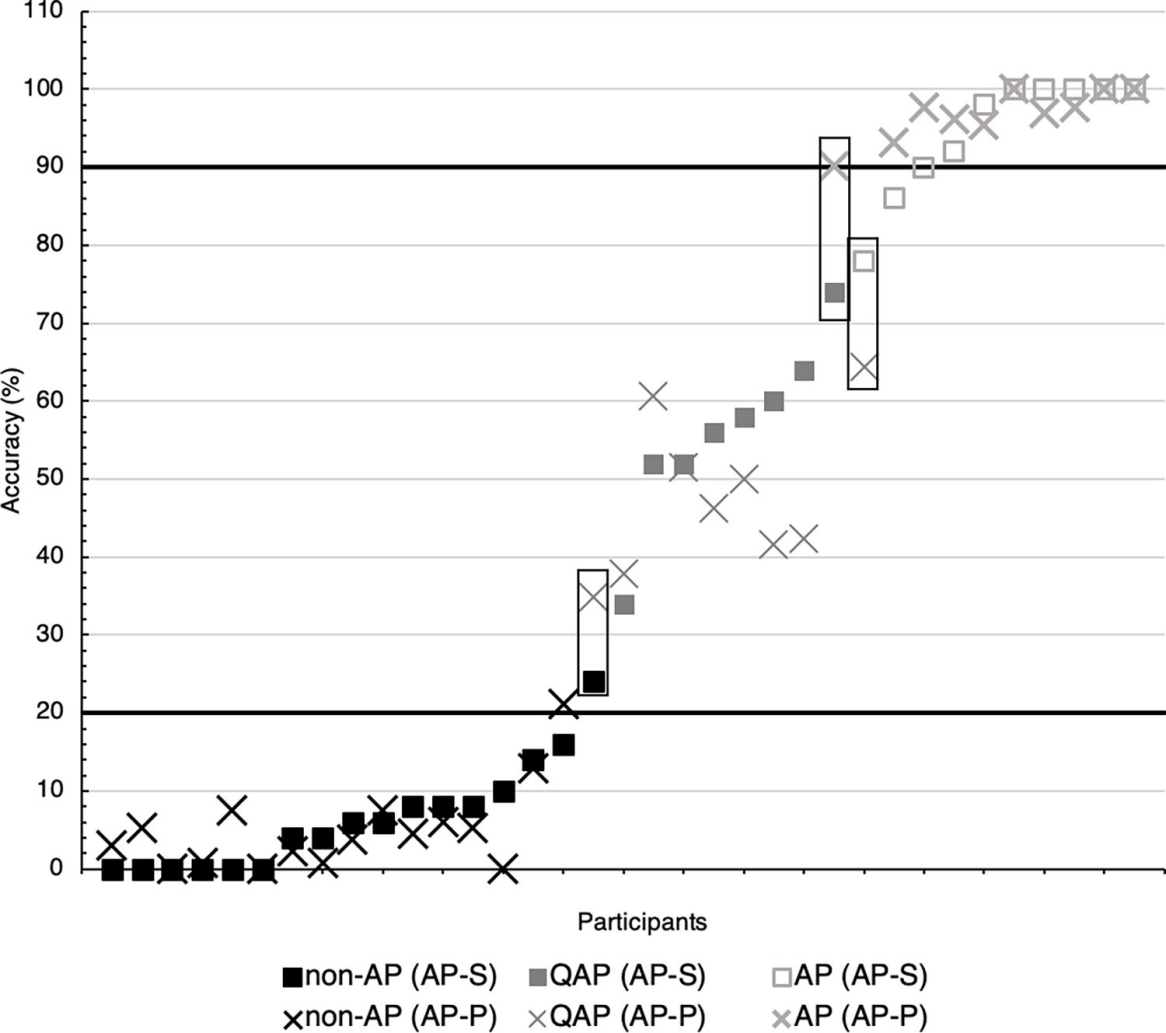
Distribution of accuracy scores. Distribution is shown across AP-S (squares) and AP-P (crosses), with groupings according to the three-group LPA solutions for each task (black = non-AP, dark grey = QAP, light grey = AP). The highlighted boxes refer to the three participants whose group allocation changed between AP-S and AP-P LPAs. For example, the participant in the right-most box scored 78% in AP-S and was allocated to the AP group, but only 64% in AP-P and was placed in the QAP group. Horizontal lines at 20% and 90% indicate commonly employed thresholds for AP (>90% accuracy) and non-AP (<20% accuracy).

Initial screening of responses for both tasks revealed no systematic pattern of errors that would indicate a mistuned AP template for any participant (such as most responses being a semitone sharp). Task accuracy for AP-P was significantly and highly correlated with AP-S, *r*(33) = .981, *p* < .001, showing that the two tasks were similar in their measurement of pitch-naming. A paired-samples t-test showed that scores did not differ significantly between the two tasks, *t*(34) = 0.526, p = .602. This is further illustrated in Fig 3, where high- and low-performing individuals scored similarly across both measures. Greater heterogeneity between task scores was evident across intermediate performers, suggesting that the use of an explicit prime affected pitch judgements for these participants.

#### AP-S group data

**Fig 4:**
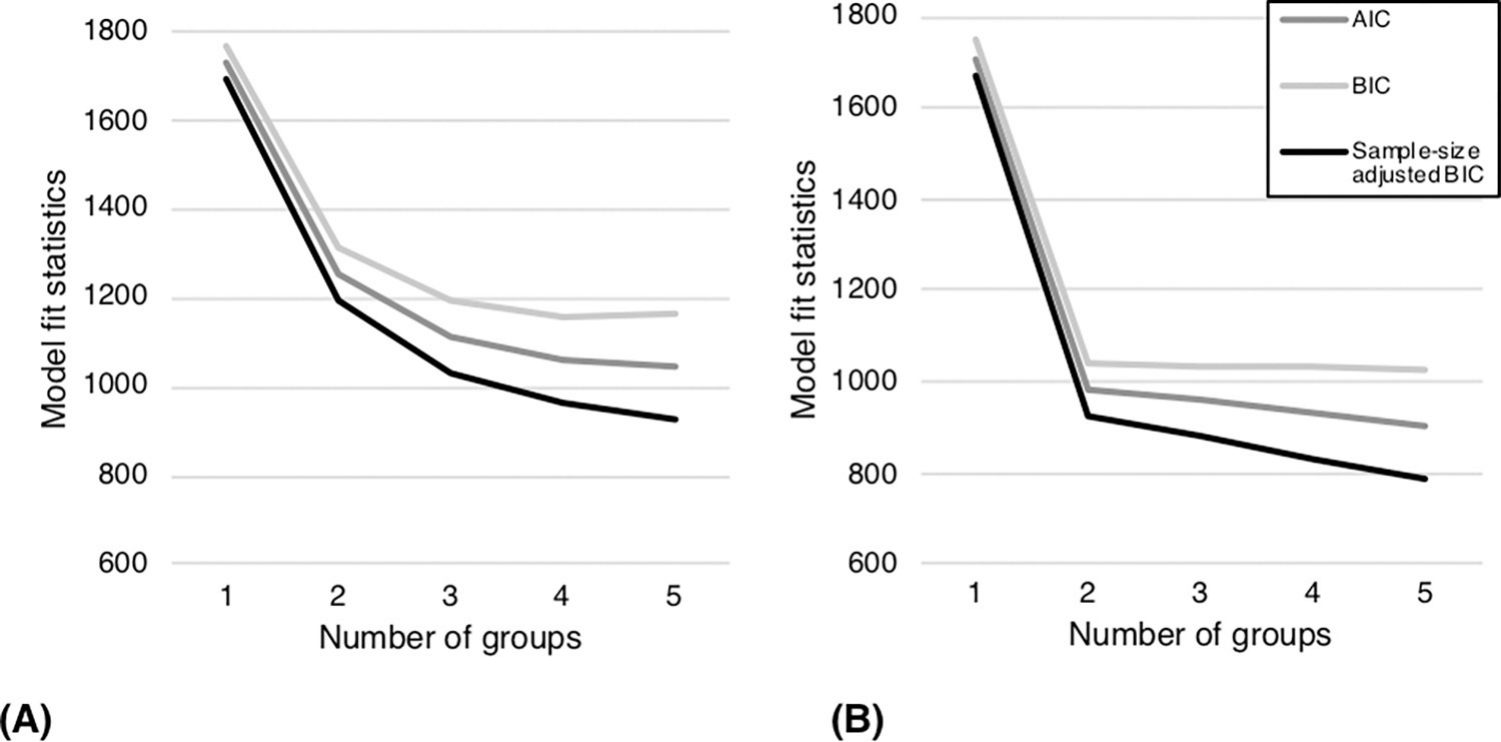
Model fit statistics for 1–5 group solutions for AP-S showing (a) AP-S chroma accuracy and (b) AP-S chroma accuracy ST.

Raw mean task accuracy for AP-S ranged from 0 to 50 (0–100%), *M* = 21.46 (42.92%), *SD* = 19.71. LPAs were conducted on AP-S chroma accuracy with the number of classes specified for each analysis ranging from one to five, to evaluate the possible number of underlying groups within the distribution. Analyses with more than five classes resulted in solutions that were untrustworthy due to local maxima. Analyses with one to five specified classes converged on a final solution with replicable log likelihood values and had appropriate levels of classification certainty (Entropy = 1). AIC, BIC, and ABIC values for one- to five-group solutions are presented in Fig 4(a). All three measures showed that the optimal way to divide participants was into either three or four groups. Class characteristics for each analysis are shown in Table 4. After four groups, the reduction in AIC, BIC, and ABIC values decreased with each additional group, yielding diminishing returns with each subsequent group division and generating groups of too few participants to be interpretable.

**Table 3:** Class characteristics in LPA models for AP-S.

Number of participants, mean AP-S accuracy (%), and range (%) per class*					
Number of classes	Class 1	Class 2	Class 3	Class 4	Class 5
1	*N =* 35 *M* = 42.92 (0–100)	-	-	-	-
2	*N* = 18 *M* = 7.89 (0–34)	*N =* 17 *M* = 80.00 (52–100)	-	-	-
3	*N =* 17 *M* = 6.35 (0–24)	*N* = 8 *M* = 56.25 (34–74)	*N =* 10 *M =* 94.40 (78–100)	-	-
4	*N* = 17 *M* = 6.35 (0–24)	N = 4 *M* = 64.00 (58–74)	*N* = 4 *M* = 48.50 (34–56)	*N* = 10 *M* = 94.40 (78–100)	-
5	*N* = 1 *M* = 24.00	*N =* 3 *M =* 47.33 (34–56)	*N* = 16 *M* = 5.25 (0–16)	*N* = 5 *M* = 61.60 (52–74)	*N* = 10 *M* = 94.40 (78–100)

*Estimated posterior probability of class membership was ≥ .999 for most likely class

Cluster analysis of AP-S chroma accuracy favoured either a two- or three-group solution, based on inspection of the dendrogram and an abrupt increase in coefficient values in the agglomeration schedule (see S1 Fig). The three-group solution was identical to the corresponding LPA solution in terms of both number of members and the individual participants comprising each group (see S1 Table). As the cluster analysis showed identical groupings to the LPA, we determined that LPAs were suitable for our dataset and would be preferable to additional cluster analyses for the reasons outlined above.

#### AP-P group data

**Fig 5:**
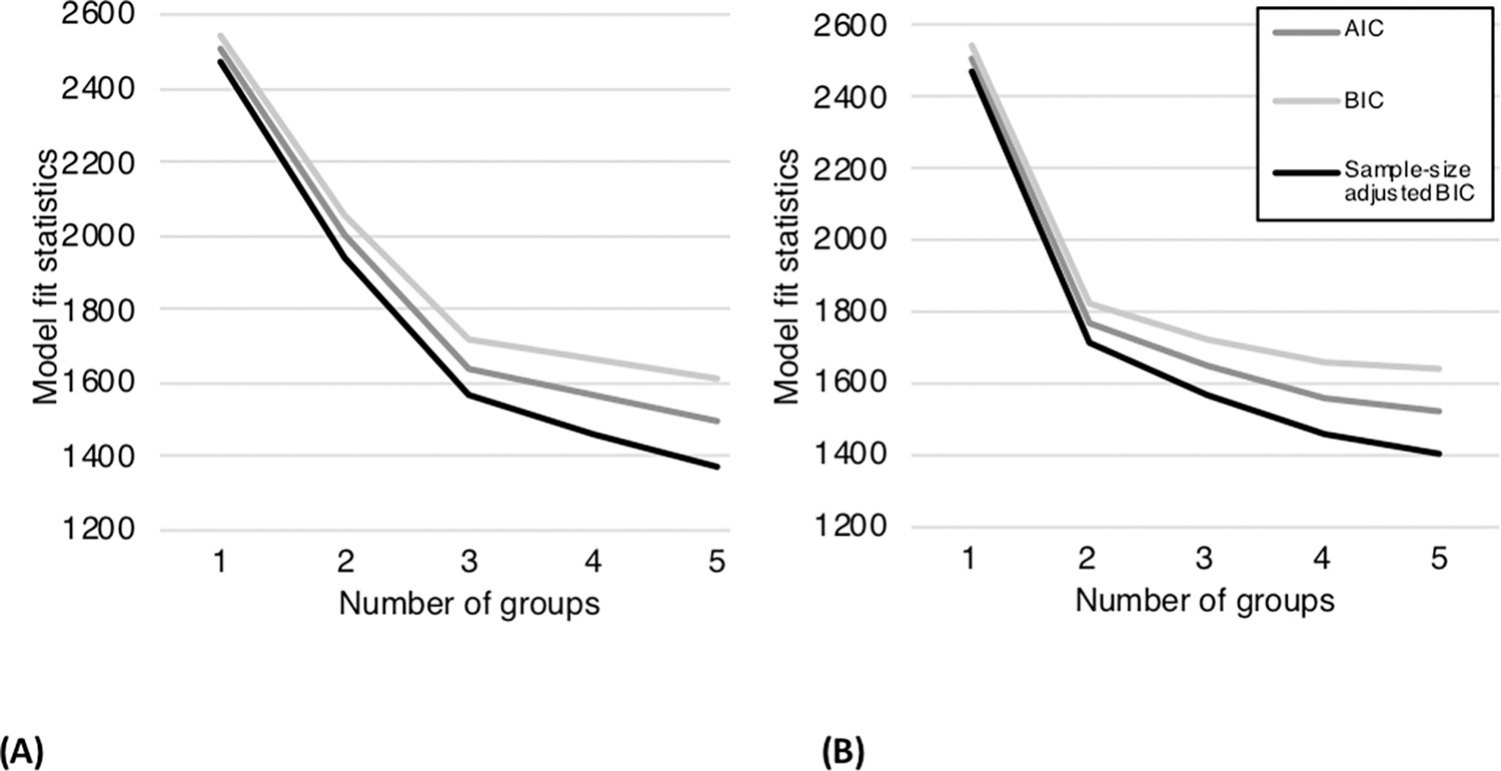
Model fit statistics for 1–5 group solutions for AP-P showing (a) AP-P chroma accuracy and (b) AP-P chroma accuracy ST.

AP-P task accuracy ranged from 0 to 132 (0–100%), *M* = 55.74 (42.23%), *SD* = 52.33. Similar LPA procedures were conducted on AP-P to AP-S, using AP-P chroma accuracy. A three-group solution was found to be optimal, using the AIC, BIC, and ABIC, as shown in Fig 5(a). Participant distributions across groups for each solution are shown in Table 4.

**Table 4:** Class characteristics in LPA models for AP-P.

Number of participants, mean AP-P accuracy (%), and range (%) per class*					
Number of classes	Class 1	Class 2	Class 3	Class 4	Class 5
1	*N* = 35 *M =* 42.23 (0–100.00)	-	-	-	-
2	*N* = 23 *M* = 16.77 (0–51.51)	*N =* 12 *M* = 91.04 (60.61–100.00)	-	-	-
3	*N* = 16 *M =* 5.07 (0–21.21)	*N =* 9 *M* = 47.73 (34.85–64.40)	*N =* 10 *M* = 96.94 (90.15–100.00)	-	-
4	*N =* 16 *M* = 5.07 (0–21.21)	*N =* 2 *M* = 56.06 (51.52–60.61)	*N =* 7 *M =* 45.35 (34.85–64.40)	*N =* 10 *M* = 96.74 (90.15–100.00)	-
5	*N =* 15 *M =* 3.99 (0–12.88)	*N =* 3 *M =* 31.31 (21.21–37.88)	*N =* 2 *M =* 56.06 (51.52–60.61)	*N =* 5 *M* = 48.94 (41.67–64.40)	*N =* 10 *M* = 96.74 (90.15–100.00)

*Estimated posterior probability of class membership = 1 for most likely class

Three intermediate-performing participants changed groups based on whether the three-group LPA solutions for the AP-S or AP-P task was used, resulting from individual score fluctuations (see Fig 3). For all subsequent analyses participants are grouped according to the AP-P-based LPA, and for consistency in nomenclature, classes based on the three groups are henceforth referred to as: Class 1 “non-AP group”, Class 2 “QAP group”, and Class 3 “AP group”.

#### AP-P individual data

For these analyses, participants who scored < 1% (*n* = 5) or at 100% (*n* = 3) on AP-P were not further assessed. For the remaining participants (*n* = 27), relative model fit of the logistic regression models was evaluated using chi-square comparisons of log likelihood values. The suitability of C as an unbiased statistical reference was verified by examining the group-level accuracy for each chroma in both the AP-S task and as primes in the AP-P task (see S3 and S4 Figs) and underscores the utility of examining participant pitch templates at an individual level. Comparing the null model against the full model with target and prime chroma, target chroma or prime chroma significantly predicted the likelihood of a correct target response for eight participants (*p* < .05). Of these, seven belonged to the QAP group (78% of QAP group) and one to the AP group (10% of the AP group). The remaining two participants in the QAP group (22%) did not have significant logistic regression models, meaning that neither target nor prime chroma identity significantly predicted target accuracy. Six participants (four QAP, one non-AP and one AP) showed significant priming effects, in that the model including the prime was a better fit than either the base model or the target-only model. Model fit statistics are shown in Table 5.

**Table 5:** Model fit statistics for individual logistic regressions for AP-P.

Participant	Group	Score (%)	Model fit statistics			
			Block 1: Target chroma as predictors		Block 2: Target & prime chroma as predictors	
			Chi square test of model fit	-2 log likelihood	Chi square test of model fit	-2 log likelihood
1	non-AP	4.55	χ^2^(11) = 11.57, *p* = .396	37.24	χ^2^(11) = 14.03, *p* = .231	23.21
5	non-AP	3.03	χ^2^(11) = 12.01, *p* = .363	23.84	χ^2^(11) = 13.40, *p* = .268	10.44
8	non-AP	2.27	χ^2^(11) = 8.53, *p* = .665	20.11	χ^2^(11) = 10.06, *p* = .525	10.04
11	non-AP	12.88	χ^2^(11) = 15.05, *p* = .180	86.35	χ^2^(11) = 24.71, ***p* = .01**^**a**^	61.64
12	non-AP	5.30	χ^2^(11) = 18.01, *p* = .081	36.73	χ^2^(11) = 15.07, *p* = .179	21.66
16	non-AP	21.21	χ^2^(11) = 14.31, *p* = .217	122.12	χ^2^(11) = 12.57, *p* = .323	109.55
18	non-AP	6.06	χ^2^(11) = 9.71, *p* = .556	50.65	χ^2^(11) = 13.24, *p* = .278	37.41
21	non-AP	5.30	χ^2^(11) = 13.77, *p* = .246	40.97	χ^2^(11) = 8.55, *p* = .663	32.42
23	non-AP	3.79	χ^2^(11) = 16.25, *p* = .132	26.30	χ^2^(11) = 20.75, ***p* = .036**^**a**^	5.55
24	non-AP	7.58	χ^2^(11) = 9.75, *p* = .553	61.08	χ^2^(11) = 13.99, *p* = .234	47.09
28	non-AP	7.58	χ^2^(11) = 13.99, *p* = .233	56.83	χ^2^(11) = 12.79, *p* = .307	44.04
6	QAP	60.61	χ^2^(11) = 61.53, ***p* < .001**	115.48	χ^2^(11) = 22.72, ***p* =** .**019**	92.76
9	QAP	41.67	χ^2^(11) = 52.95, ***p <* .001**	126.35	χ^2^(11) = 16.80, *p* = .114	109.56
10	QAP	46.21	χ^2^(11) = 19.79, ***p* = .048**	162.45	χ^2^(11) = 30.10, ***p* = .002**	132.34
15	QAP	64.39	χ^2^(11) = 18.84, *p* = .064	153.06	χ^2^(11) = 33.75, ***p* < .001**	119.31
26	QAP	51.52	χ^2^(11) = 76.64, ***p* < .001**	106.23	χ^2^(11) = 13.85, *p* = .241	92.38
27	QAP	42.42	χ^2^(11) = 15.52, *p* = .160	164.43	χ^2^(11) = 34.71, ***p* < .001**	129.72
32	QAP	34.85	χ^2^(11) = 38.14, ***p* < .001**	132.54	χ^2^(11) = 11.74, *p* = .384	120.80
36	QAP	37.88	χ^2^(11) = 14.38, *p* = .213	160.78	χ^2^(11) = 14.49, *p* = .207	146.29
37	QAP	50.00	χ^2^(11) = 15.57, *p* = .158	167.42	χ^2^(11) = 10.18, *p* = .514	157.24
4	AP	93.18	χ^2^(11) = 20.75, ***p* = .036**^a^	44.96	χ^2^(11) = 16.55, *p* = .122	28.41
7	AP	95.45	χ^2^(11) = 15.82, *p* = .148	33.00	χ^2^(11) = 13.93, *p* = .237	19.07
14	AP	96.97	χ^2^(11) = 9.04, *p* = .618	26.81	χ^2^(11) = 15.34, *p* = .168	11.47
20	AP	96.21	χ^2^(11) = 12.00, *p* = .363	30.36	χ^2^(11) = 16.62, *p* = .120	13.92
30	AP	97.73	χ^2^(11) = 8.53, *p* = .665	20.11	χ^2^(11) = 11.03, *p* = .441	9.08
31	AP	90.15	χ^2^(11) = 21.16, ***p* = .032**	63.78	χ^2^(11) = 28.16, ***p* = .003**	35.62
33	AP	97.73	χ^2^(11) = 8.53, *p* = .665	20.11	χ^2^(11) = 12.47, *p* = .329	7.64

a: overall model significant, but no individual chroma emerged as predictors

Participants omitted from this table: 2, 34, 35 (100% accuracy on priming task); 13, 19, 22, 25, 29 (< 1% accuracy on priming task). Participants 3 and 17 did not complete the experimental protocol.

**Fig 6:**
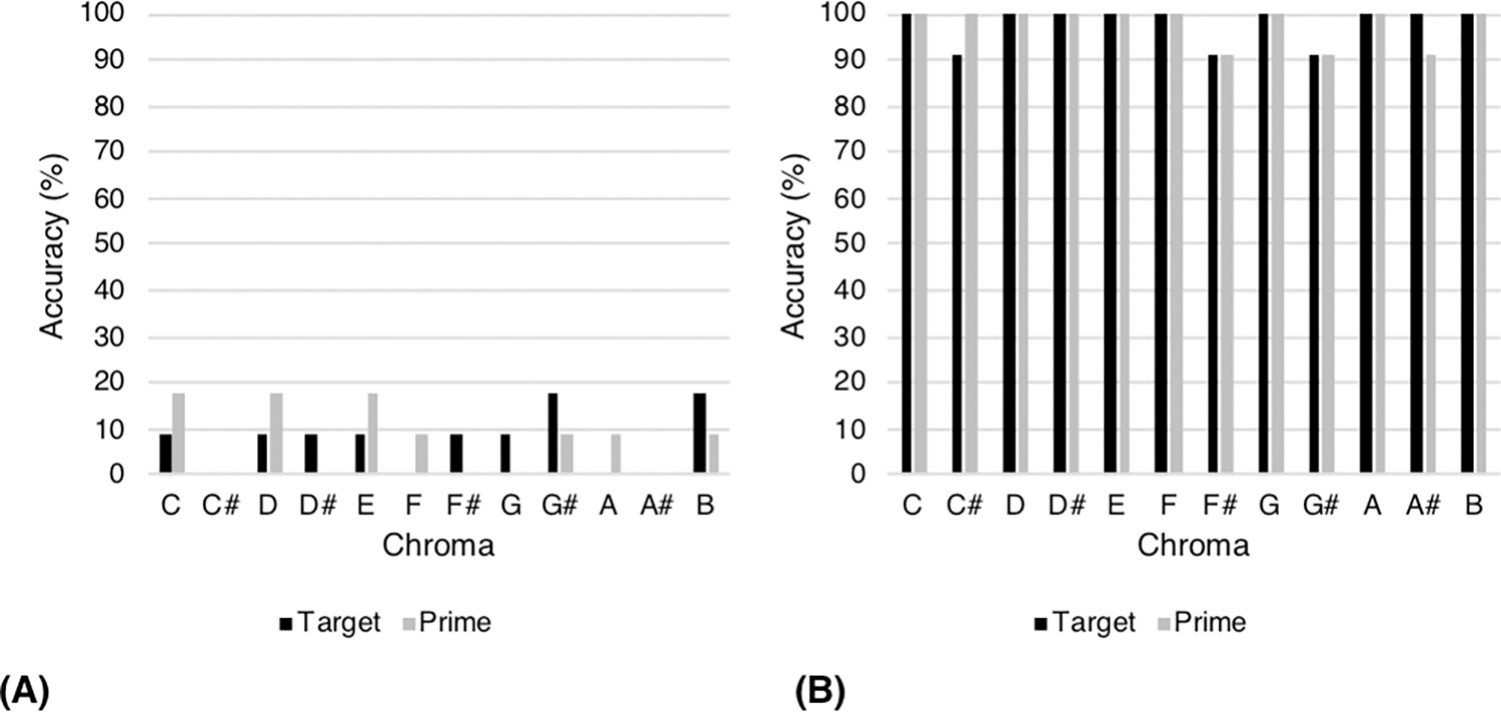
Accuracy profiles for a typical (a) non-AP participant (Participant 24; AP-P task accuracy 7.58%) and (b) AP participant (Participant 30; AP-P task accuracy 97.73%). Target bars refer to the percentage of those trials for which each chroma was a target that was correctly identified by the participant. Prime bars refer to the percentage of those trials for which each chroma was a prime that preceded a correct target identification by the participant.

Fig 6 shows the typical chroma accuracy profiles for the remaining non-AP and AP participants. Neither target chroma nor prime chroma predicted the likelihood of a correct response as these participants tended to perform near chance or ceiling, respectively.

**Fig 7:**
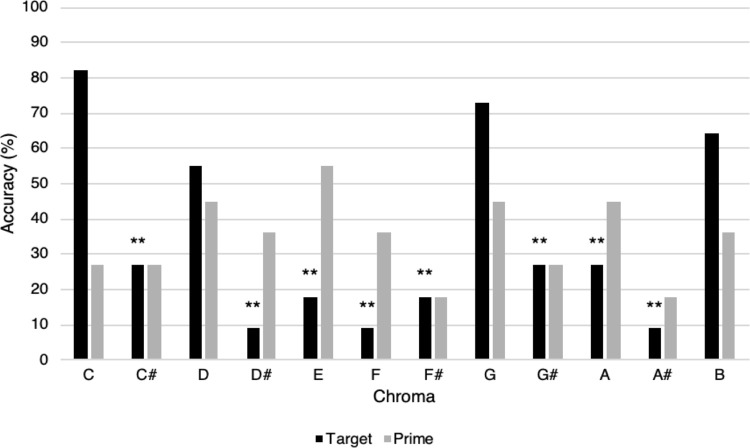
Accuracy profile for a QAP participant (participant 32) with significant target chroma, AP-P task accuracy 34.85%. Marked target chroma were significantly less likely to be accurately identified than the statistical reference point of C, according to this participant’s logistic regression model. As the majority of “poor” target chroma corresponded to the black keys of the piano, this participant may be considered to have “white-note” AP (relatively good performance for white-key chroma). As prime chroma did not significantly predict the likelihood of a correct response, it is unlikely that this participant employed a reference chroma strategy for identifying pitches (**p < .01).

**Fig 8:**
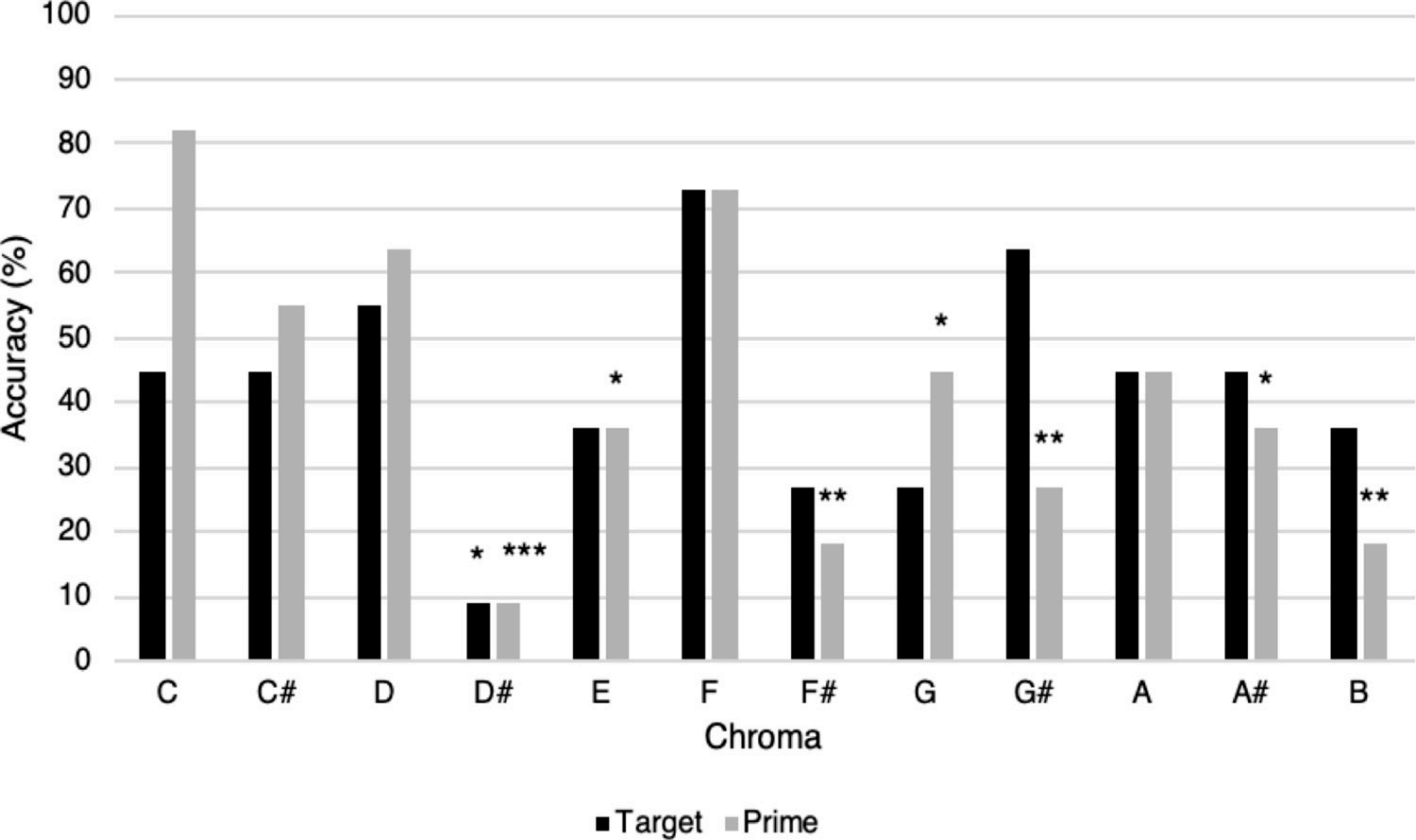
Accuracy profile for a participant (participant 27) with significant target and prime chroma, AP-P task accuracy 42.42%. Marked target chroma were significantly less likely to be accurately identified than the statistical reference point of C, according to this participant’s logistic regression model. Marked prime chroma were significantly less likely to precede an accurately identified target than targets following C primes (*p < .05, **p < .01, ***p < .001).

Figs 7 and 8 illustrate chroma accuracy profiles for two QAP participants (for additional accuracy profiles, see S5–S10 Figs). For participant 32 (Fig 7), target chroma significantly predicted the likelihood of a correct response, such that several chroma were less likely than C to result in correct target identification. No significant primes were identified. For participant 27 (Fig 8), significant differences among chroma were identified for both targets and primes, such that both target chroma and prime chroma predicted the likelihood of a correct response. In general, the pattern of significant chroma was variable among QAP participants, however notes corresponding to the white keys of the piano were more frequently strong targets/primes compared to black key notes.

### Factors associated with AP ability

Demographic and musical experience data for all participants are shown in Table 6, along with results of analyses. Participants are grouped according to the AP-P-based LPA. A Box-Cox transformation (λ = -0.1) was applied to the Age variable to adjust for skew. For categorical variables, differences between groups were found for playing a fixed pitch main instrument (AP = QAP > non-AP), being of Asian ethnic background (AP > QAP > non-AP, and having a family history of AP (AP > QAP > non-AP), while the remaining variables showed no significant effects (*p* > .05).

**Table 6:** Demographic and musical experience differences between groups.

Variable	Non-AP (*n* = 16)	QAP (*n* = 9)	AP (*n* = 10)	Statistical test value	Significance
Categorical variables (n, %)				Fisher’s Exact Test	
Male	6 (37.5%)	5 (55.56%)	3 (30%)	1.37	0.558
Asian ethnic background	2 (12.5%)	2 (22.22%)	7 (70%)	9.77	0.015*
First language tone language	1 (6.25%)	1 (11.11%)	4 (40%)	4.48	0.086
Fixed do music training	4 (25%)	2 (22.22%)	6 (60%)	3.80	0.133
Main instrument fixed pitch	9 (56.25%)	9 (100%)	10 (100%)	9.03	0.005**
First instrument fixed pitch	12 (75%)	9 (100%)	9 (90%)	2.57	0.346
Family history of AP	0 (0%)	1 (11.11%)	3 (30%)	4.95	.048*
Continuous variables (mean, range)				One-Way ANOVA	
Age (years)	32.13 (19–54)	32.11 (18–68)	20.90 (18–26)	*F*(2, 32) = 5.47^a^	.009**
Age commenced music training (years)	6.63 (3–11)	5.56 (3–10)	4.20 (3–7)	*F*(2, 32) = 4.63	.017*
Length of music training (years)	7.72 (0.5–15)	13 (4–20)	14 (9–18)	*F*(2, 32) = 10.37	< .001***
Length of music theory training (years)	2.50 (0–15)	7.56 (0–14)	6.40 (1–15)	*F*(2, 32) = 8.08	.001**
Current music practice (hours per week)	5.69 (0–20)	11.11 (2–21)	11.20 (5–22)	*F*(2, 32) = 3.39	.046*
Current music listening (hours per week)	22.50 (3–70)	15.89 (4–42)	18.80 (4–50)	*F*(2, 32) = 0.432	.653

* *p* < .05

** *p* < .01

*** *p* < .001

^a^ANOVA performed on Box-Cox transformed Age variable

As shown in Table 6, for continuous variables differences between the groups were found for age (AP < QAP = non-AP), age of commencement of music training (AP < QAP < non-AP), years of music training (AP > QAP > non-AP), years of music theory training (AP < QAP > non-AP), and current hours of music practice (AP = QAP > non-AP). There were no other significant differences between the groups (*p* > .05). Two older QAP participants contributed disproportionately to the age effect, with the remaining QAP participants below 30 years of age. When we removed these older, outlying participants, the non-AP group became the oldest of the three groups, but no other continuous or categorical effects were changed. We therefore retained the complete QAP group for our analyses.

Summarising the above findings, the majority of differences occurred between the AP and non-AP groups, with QAP values falling in-between these groups. The QAP group was similar to the AP group for playing a fixed pitch main instrument, length of music and theory training and current hours of music practice, but closer in age to the non-AP group. AP possessors tended to be younger than non-AP and QAP individuals, were more likely to have an Asian ethnic background, and commenced music training at a younger age. Non-AP participants had fewer years of music training (including music theory training), were less likely to play a fixed pitch instrument (piano) as their main instrument, practised music less frequently, and had no family history of AP.

### Impact of scoring methods

Mean AP-S task accuracy ST was 27.40 (54.80%), *SD* = 20.07. LPAs were conducted as described for the raw accuracy scores, with solutions converging for up to five groups. Classification quality was appropriate for all models, with entropy ≥ .998. Model fit changes for the AIC, BIC, and ABIC are shown in Fig 4(b). Based on the AIC, BIC, and ABIC, a two-group solution was preferred for data where semitone errors were counted as correct.

Mean AP-P task accuracy ST was 72.86 (55.20%), *SD* = 53.05. LPAs were run for AP-P target chroma accuracy ST, also resulting in a two-group solution consisting of ceiling and floor performance groups as shown in Fig 4(b).

## Discussion

In this study, we applied a novel approach to investigating AP in order to characterize variability in pitch-naming ability. We found that participant accuracy on an AP priming task was highly correlated with accuracy on a standard AP task, supporting the utility of this novel explicit priming task as a test of pitch-naming ability. A key contribution of our preliminary study is the demonstration that priming is useful in exploring the pitch templates of QAP participants, allowing us to identify phenotypic variability in AP and explore the contributions of RP processing in those with intermediate pitch-naming ability. Our findings show that the priming task can (i) differentiate between intermediate performers who do (4/9; 44%) or do not (5/9; 56%) benefit from a priming effect (via a RP strategy), and (ii) reliably identify high-accuracy (AP) and chance-level (non-AP) performers in line with a standard pitch-naming task.

Supporting our prediction of differing AP phenotypes, we found evidence of three levels of ability within our sample, using both hierarchical cluster analysis and LPA. Across both the AP-S and AP-P tasks, LPAs reliably divided participants into clearly defined AP and non-AP groups, with a heterogeneous class of intermediate performers corresponding to a QAP group. As the number of QAP musicians in our sample is limited, however, any additional subgroups could not be further explored, warranting further investigation in a larger sample. Due to the preliminary nature of this study, we do not claim that the three-group solution is necessarily the optimal division of ability levels, rather our primary focus was to investigate the utility of the priming task itself.

Our initial findings, however, argue against the classification of AP as a categorical ‘all-or-nothing’ trait, as shown by the lack of support for a two-group solution in the LPAs. The question of whether AP consists of more than two distinct phenotypes or is the endpoint of a continuum of ability has not been addressed here, and it should be highlighted that the notion of QAP as an extension of AP, rather than a distinct type of pitch-naming ability, has recently been supported by Van Hedger and colleagues [21] using data-driven methods. Of note, our distribution of ability appears consistent with either three categories (as per the LPA), or a continuum represented by a sigmoid function (see S2 Fig). The suitability of each classification approach should be investigated in a larger sample through taxometric analysis [87].

The data-driven approach of LPA is an improvement on typical AP research methods of arbitrarily defined category boundaries. Different AP studies have not been comparable due to different cut-offs for assigning AP status, such as 80% [88] versus 90% with semitone errors [35], potentially conflating phenotypically distinct groups. Replication of LPAs with larger samples may identify optimal thresholds for reliably identifying AP phenotypes. It should be noted that LPAs are generally recommended for use with larger samples–however, as an initial analysis, we have shown the utility of such an approach with AP data, and the generated groups aligned with those produced by traditional hierarchical cluster analysis. Furthermore, the highly correlated distributions of the AP-S and AP-P tasks provide important validation for AP-P as a measure of pitch-naming ability.

Having established a QAP group using both the AP-S and AP-P tasks, we further investigated the heterogeneity in this group through analysis of individual participant pitch templates as revealed by the AP-P task. These analyses showed which chroma were more likely to be correctly identified by each participant, and also which chroma were likely to serve as effective primes facilitating a correct target response. The AP-P task is unique in that it enables the observation of a cognitive process often reported by musicians, but not previously observed experimentally. The use of reference chroma has been cited as a defining feature of QAP musicians [13] and is potentially facilitated by pitch working memory during pitch-naming tasks [20], yet the few studies investigating QAP have relied on musician self-report of this strategy. Although we have a small number of QAP musicians, heterogeneity of strategy use is apparent in examining response patterns to AP-P, with no chroma serving as a universally beneficial prime. Future research can incorporate self-report measures to examine participants’ perceptions of the strategies they employ. Self-report items would explore whether participants’ perceptions of their “best” chroma are reflected in their performance, potentially accounting for variance in the QAP data.

Some QAP participants showed no prime or target effects (*n* = 2), while others had clearly preferred targets and primes. For example, participant 27 (see Fig 8) appeared heavily reliant on prime chroma for target identification, suggesting frequent use of a reference tone strategy. When the prime was a chroma in the participant’s pitch template (in this example, C in particular), this was likely to lead to a correct response regardless of target chroma identity. From this, we can conclude that the participant likely used RP strategies to correctly deduce the target chroma, as per the RP decision process in Fig 2. If, however, the prime was not in the participant’s pitch template, this was likely to lead to an incorrect response to the target as an RP strategy could not be appropriately used. Contrary to our expectations, the presence of two stimuli was sometimes disruptive rather than facilitative or neutral (see the participants whose AP-P score was poorer than their AP-S score in Fig 3). If a participant’s internal representation of the prime was not sufficiently stable, this may have interfered with attempts to identify the chroma of the target stimulus. For example, despite a strong prime effect for C for participant 27, this was not reflected in the target accuracy for C (only 45% of trials in which C was a target were correctly identified). This suggests that the internal representation of C in this participant’s pitch template may have been disrupted when paired with less stable primes (for example, D♯ and F♯), but was sufficiently stable to facilitate accurate target identification when presented as the initial stimulus (prime). Similarly, misidentification of a less stable prime (for example, labelling an F prime as an F♯) would lead to incorrect target identification even if RP strategies are used. Although this requires further exploration, a modification to the “guessed response” process in Fig 2 may be “Neither C nor G are *stable* in pitch template.”

Other QAP participants (such as participant 32, see Fig 7) correctly identified chroma within their pitch templates, but did not appear to use RP strategies to facilitate pitch naming, as the identity of the prime was unrelated to the likelihood of a correct response to the target chroma. The AP-P task may therefore provide a useful means of systematically identifying individual differences in the pitch templates of QAP musicians that can only be inferred from reaction time measures in standard AP tasks. Moreover, systematic use of reference tones may constitute only one type of QAP phenotype. In a larger sample, we may be able to observe further prime-dependent and prime-independent subgroups with QAP pitch templates, and explore the potential effects of timbre and pitch range. As an initial foray into this method, the present results are promising.

In our exploratory analysis of factors linked to AP possession, expected demographic and musical experience factors broadly discriminated between our generated phenotypes. However, as this was not the primary aim of our study, and LPA-generated groups were small, definitive conclusions cannot be drawn from these findings. Rather, these analyses serve as a preliminary validation measure for our LPA classification, as the phenotypes share a pattern of predisposing factors found in previous research on AP possessors [7, 33, 50, 51, 89–95]. In particular, differences were found for age, duration of music training (including training in music theory), age of commencement of music training, hours of current music practice, having a family history of AP, and Asian ethnicity. These differences require exploration in a larger sample to more comprehensively show how demographic variables and music experience vary along the AP spectrum.

As a demonstration of the impact of scoring choices on generated phenotypes, we assigned credit to semitone errors in AP-S and AP-P. This masked sufficient variability in the sample that a two-group solution emerged for both tasks, consistent with previous reports of an AP dichotomy. The participants in the initial QAP group had sufficient responses within one semitone of the correct chroma that they merged into the AP group when these errors were assigned credit. Although this is apparent from a statistical standpoint–reducing variability in a sample will result in a smaller number of generated groups–it is conceptually important for AP research, particularly as the field moves towards identifying genetic variants that may underpin the skill. Phenotypically, there is a difference between a participant who consistently identifies every chroma correctly, and one who has reasonably high accuracy yet frequently makes small errors. Assigning credit to semitone errors conflates these two groups and thus reduces our ability to precisely phenotype AP, highlighting the impact of different approaches to scoring AP performance. More exact phenotyping will also benefit future endeavours to potentially identify underlying genetic variants.

## Conclusion

Our study has shown that consideration of phenotypes is paramount in investigations of AP, highlighting the necessity for robust methods to ensure that the phenomena revealed are representative of true phenotypic differences. For the first time, we have been able to show how RP strategies can be used to bolster incomplete pitch-naming templates through use of an AP priming paradigm, revealing individual differences within a QAP group. While our results are preliminary, they point to the importance of exploring individual pitch templates and subgroups, particularly within a QAP group. This study represents a considerable step forward in AP research, furthering our understanding of this trait and facilitating future research into its underlying mechanisms.

## Supporting information

S1 Table Participant-level composition of LPA-generated groups. (TIF)

S1 Fig Cluster analysis showing participant groupings for AP-S task. The dendrogram in (A) shows the stepwise merging of groups from 35 individuals (leftmost) to a single large group (rightmost). Note the grouping of participants into three groups, indicated by circled participant numbers. The solid circle is AP-S group 3 (AP); the dashed circle is AP-S group 2 (QAP); and the dotted line encloses AP-S group 1 (non-AP). These groups correspond to the three-group solution for the AP-S LPA and have the same members (see S1 Table). The dendrogram in (B) shows an abrupt increase at the second-last merge (circled), indicating an improvement in model fit when transitioning from four groups to three groups.
(TIF)

S2 FigThis shows the fit of a continuous sigmoid function to the distribution of AP-S scores.The function is of the form $y=\frac{a}{1+e^{-b(x-c)}}$ The fit of this function raises the possibility that pitch-naming ability is a dimensional trait rather than comprising discrete categories such as AP, QAP and RP.(TIF)

S3 Fig Mean chroma accuracy in the AP-S task for the 27 participants included in the logistic regression analyses. Error bars represent standard errors of the mean. Scores were higher for white key chroma than black key chroma, *t*(322) = 2.96, *p* = .003. Among the white key chroma, no chroma significantly outperformed any other, *F*(6, 182) = 0.163, *p* = .986.
(TIF)

S4 Fig Mean prime accuracy for the AP-P task for the 27 participants included in the logistic regression analyses. Each bar represents the mean accuracy for trials in which the relevant chroma was a *prime*. Error bars represent standard errors of the mean. Accuracy for white key chroma did not significantly differ from black key chroma, *t*(322) = 1.54, *p* = .124.
(TIF)

S5 Fig For Participant 6, E and F were well-represented in the pitch template and may have been used as reference tones for identifying other chroma, as indicated by the significance of these chroma as primes. F♯ and A♯ were rarely identified accurately as targets regardless of the preceding prime.
(TIF)

S6 Fig Participant 9 performed relatively well if targets were C, C♯, D, F, G♯ or A, but struggled to identify other target chroma. C♯ may have been a facilitative prime, but overall this participant did not appear to make use of reference tones.
(TIF)

S7 Fig Participant 10 identified D♯ targets significantly more often than C targets, although D♯ was a particularly poor prime (along with G♯). The discrepancy between D♯ as a target and a prime may suggest that the participant effectively used RP strategies to identify D♯ targets from their preceding primes, but that a mis-identified D♯ prime led to poor target identification through mistakenly applied RP strategies.
(TIF)

S8 Fig Participant 15 did not appear to make use of the primes as reference tones, as shown by the lack of significant differences among the prime chroma. E, F♯, and A were less strongly represented in the pitch template than C.
(TIF)

S9 Fig Participant 26 also did not appear to make use of primes. Note that this participant shows a clear white-key preference, with black-key targets more frequently misidentified. Significantly poor target chroma were C♯, D♯ F♯, G♯, and A♯.
(TIF)

S10 Fig Participant 31 accurately identified most chroma, but targets following a D prime were correctly identified significantly less often than C. The D prime may have had a disruptive effect for this participant.
(TIF)
