# Supplementary figures and images for: Use of explicit priming to phenotype absolute pitch ability
Source: PLoS One. 2022 Sep 14;17(9):e0273828. doi: 10.1371/journal.pone.0273828 (PMC9473427; doi:10.1371/journal.pone.0273828)

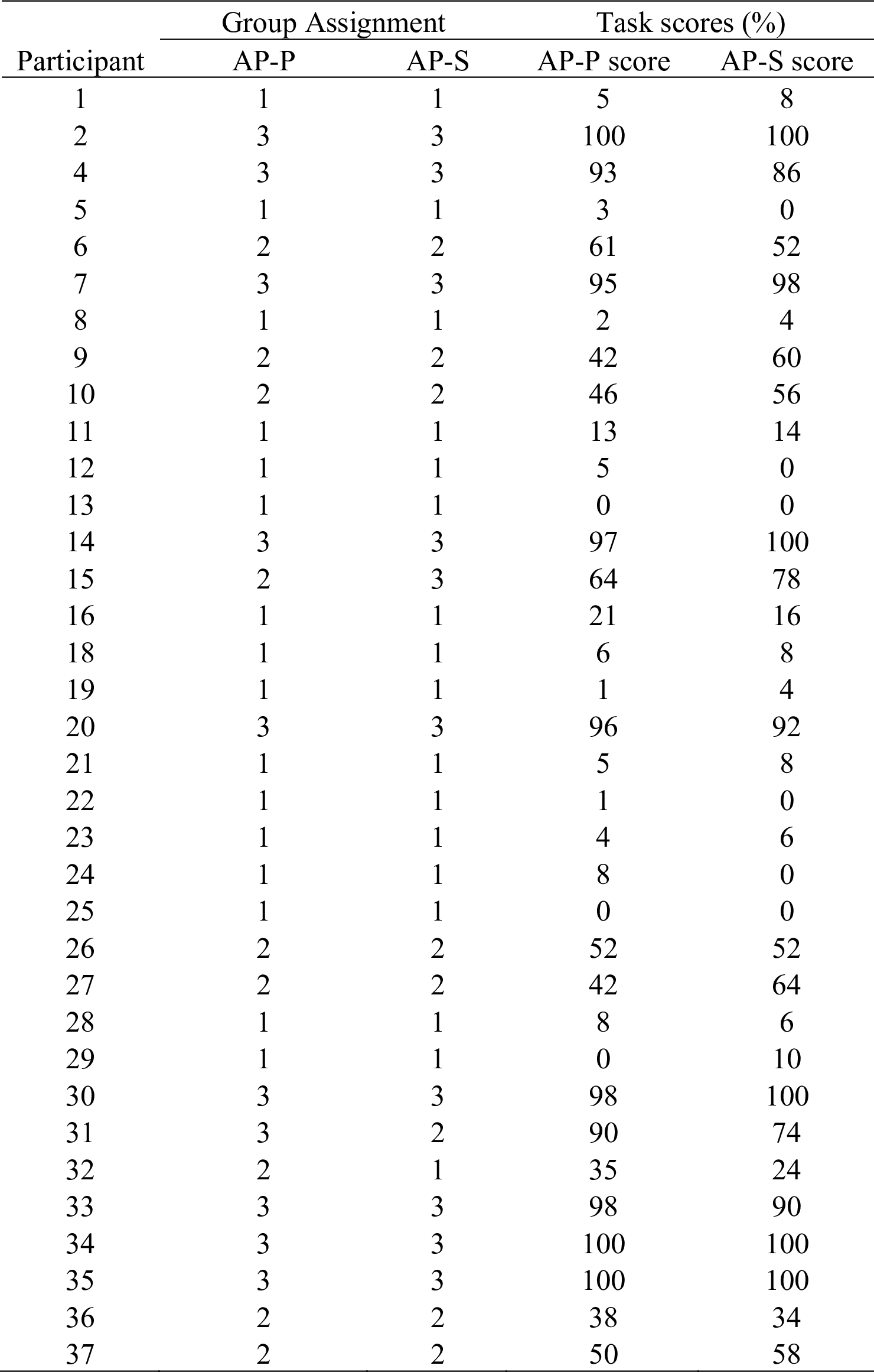

Supplement: S1 Table — (TIF) [file pone.0273828.s001.tif]

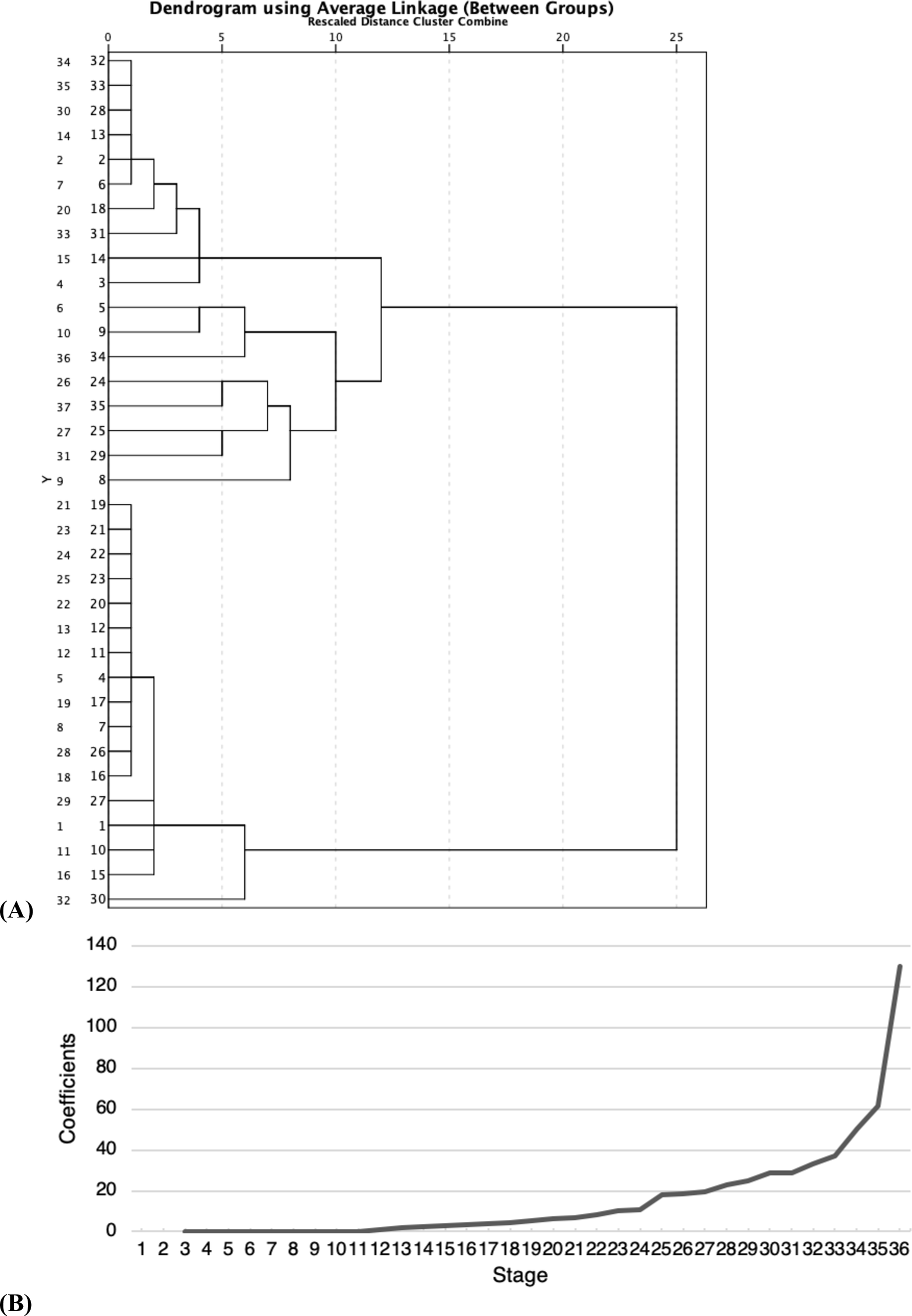

Supplement: S1 Fig — The dendrogram in (A) shows the stepwise merging of groups from 35 individuals (leftmost) to a single large group (rightmost). Note the grouping of participants into three groups, indicated by circled participant numbers. The solid circle is AP-S group 3 (AP); the dashed circle is AP-S group 2 (QAP); and the dotted line encloses AP-S group 1 (non-AP). These groups correspond to the three-group solution for the AP-S LPA and have the same members (see S1 Table). The dendrogram in (B) shows an abrupt increase at the second-last merge (circled), indicating an improvement in model fit when transitioning from four groups to three groups. (TIF) [file pone.0273828.s002.tif]

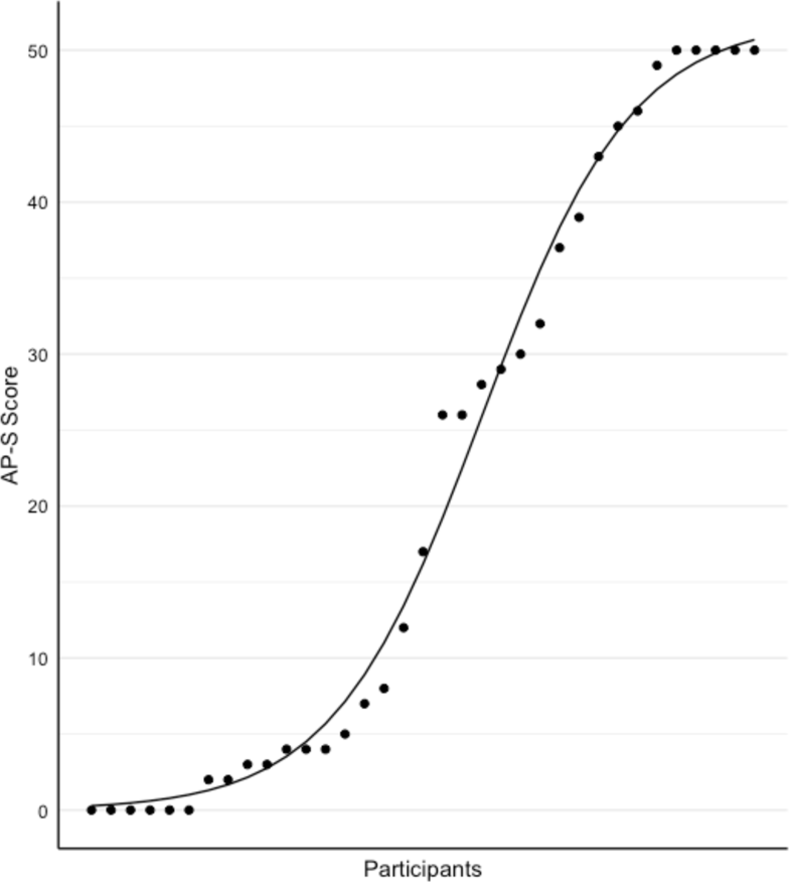

Supplement: S2 Fig — The function is of the form y=a1+e−b(x−c) The fit of this function raises the possibility that pitch-naming ability is a dimensional trait rather than comprising discrete categories such as AP, QAP and RP. (TIF) [file pone.0273828.s003.tif]

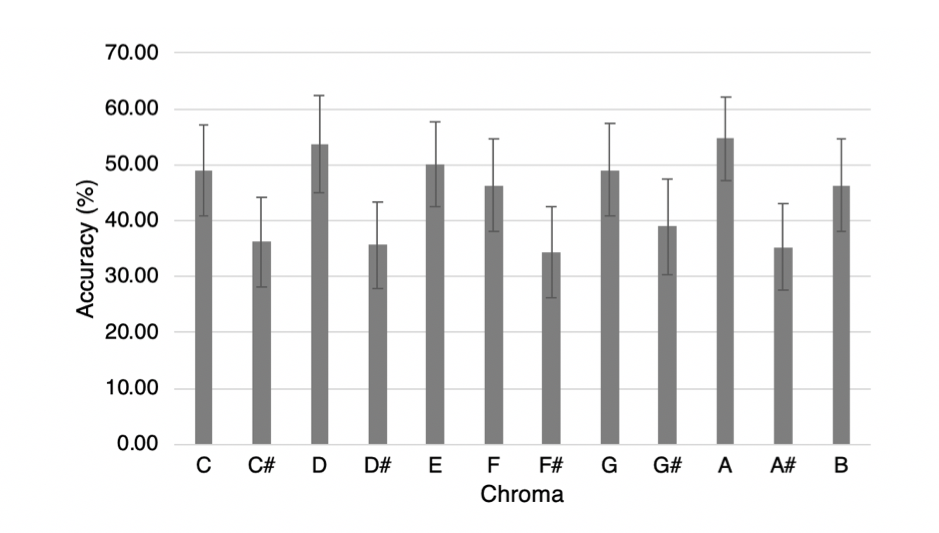

Supplement: S3 Fig — Error bars represent standard errors of the mean. Scores were higher for white key chroma than black key chroma, t(322) = 2.96, p = .003. Among the white key chroma, no chroma significantly outperformed any other, F(6, 182) = 0.163, p = .986. (TIF) [file pone.0273828.s004.tif]

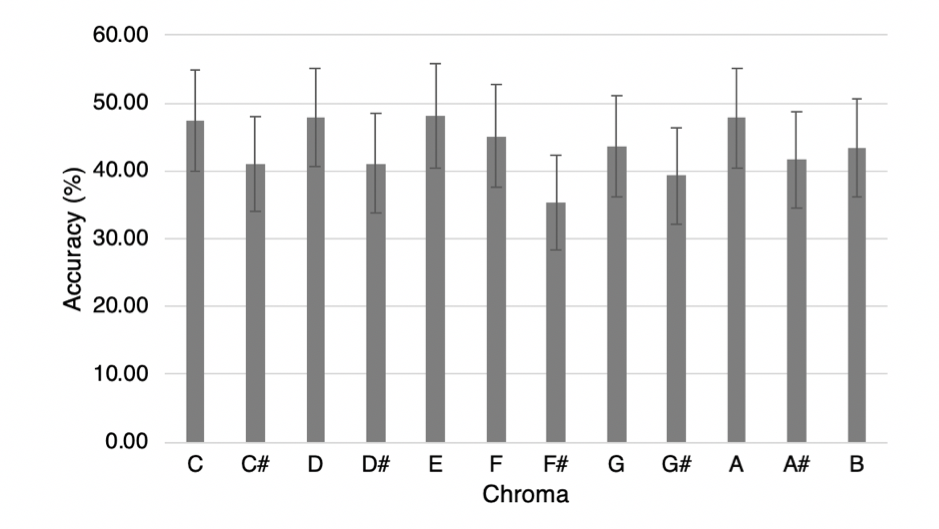

Supplement: S4 Fig — Each bar represents the mean accuracy for trials in which the relevant chroma was a prime. Error bars represent standard errors of the mean. Accuracy for white key chroma did not significantly differ from black key chroma, t(322) = 1.54, p = .124. (TIF) [file pone.0273828.s005.tif]

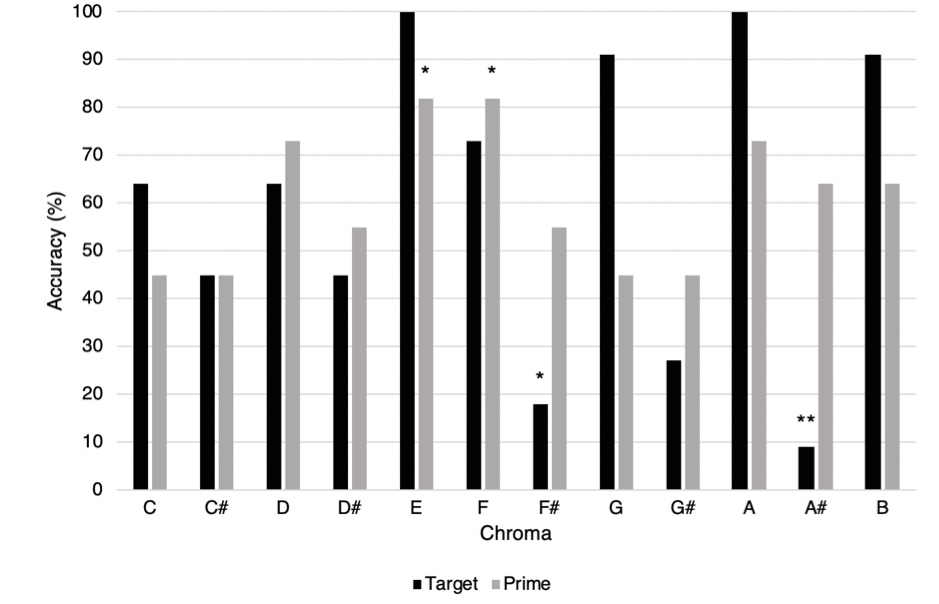

Supplement: S5 Fig — F♯ and A♯ were rarely identified accurately as targets regardless of the preceding prime. (TIF) [file pone.0273828.s006.tif]

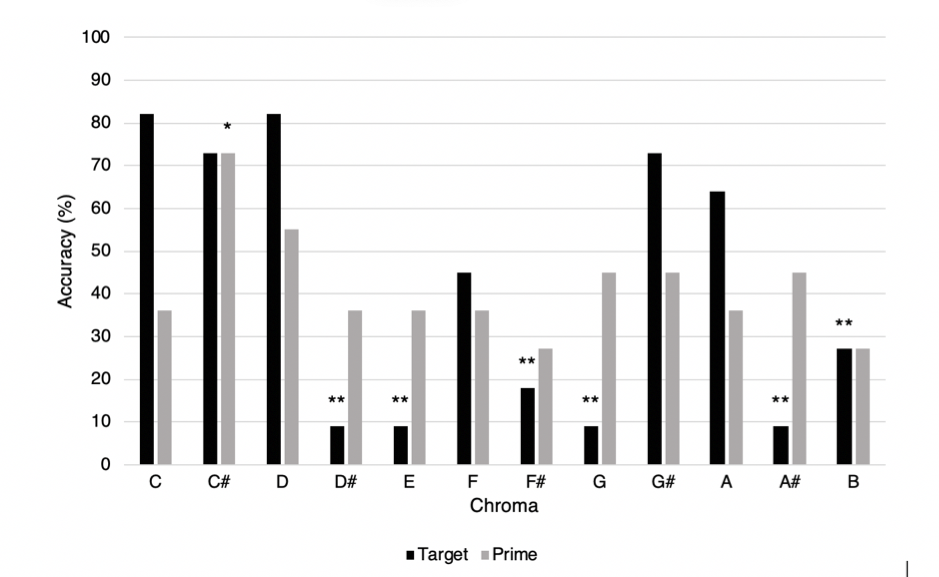

Supplement: S6 Fig — C♯ may have been a facilitative prime, but overall this participant did not appear to make use of reference tones. (TIF) [file pone.0273828.s007.tif]

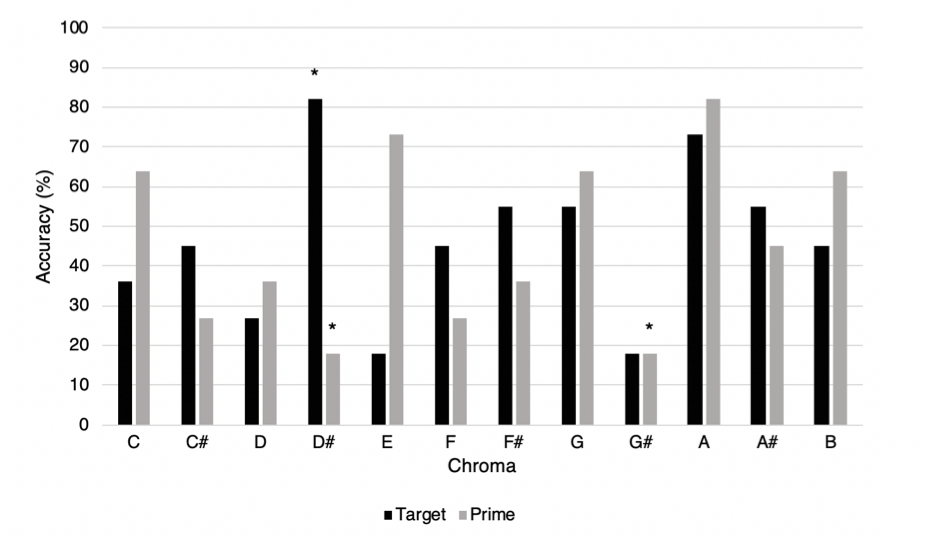

Supplement: S7 Fig — The discrepancy between D♯ as a target and a prime may suggest that the participant effectively used RP strategies to identify D♯ targets from their preceding primes, but that a mis-identified D♯ prime led to poor target identification through mistakenly applied RP strategies. (TIF) [file pone.0273828.s008.tif]

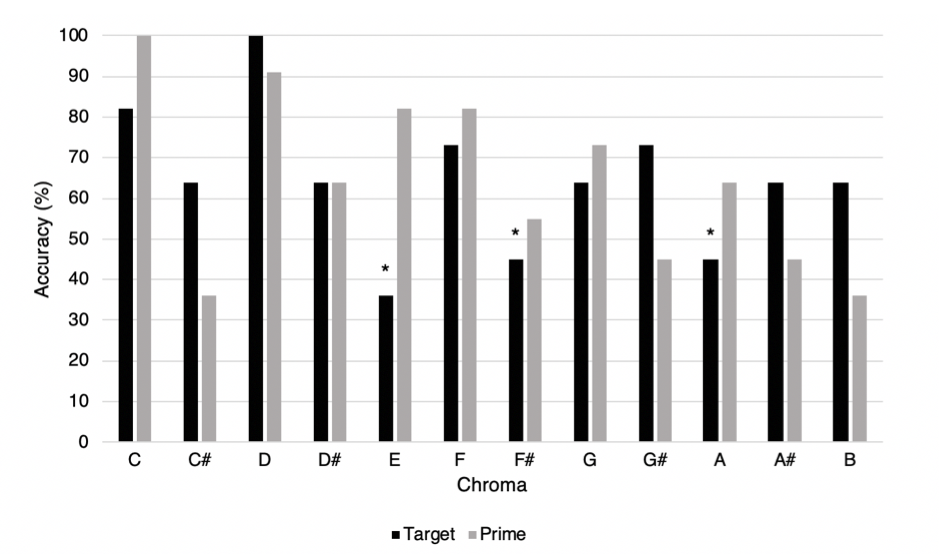

Supplement: S8 Fig — E, F♯, and A were less strongly represented in the pitch template than C. (TIF) [file pone.0273828.s009.tif]

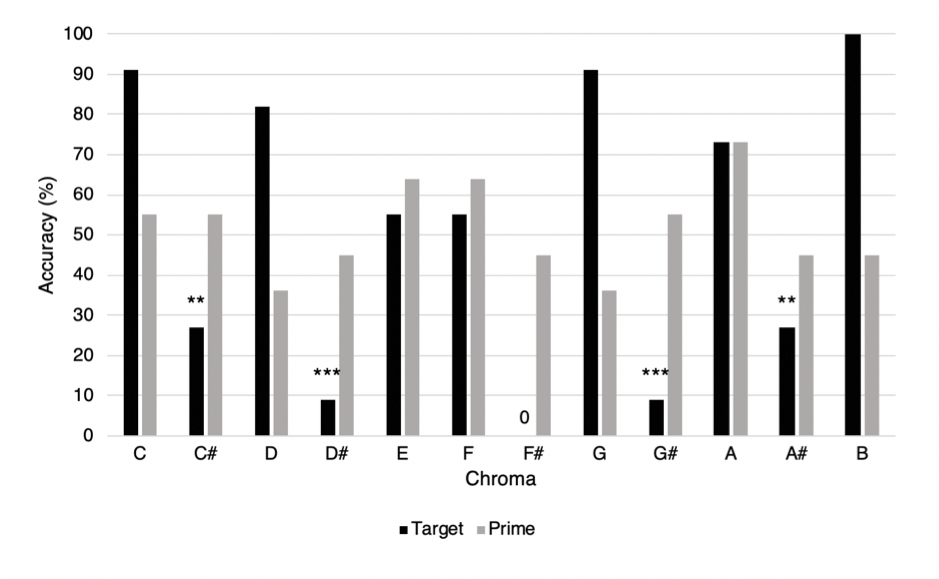

Supplement: S9 Fig — Note that this participant shows a clear white-key preference, with black-key targets more frequently misidentified. Significantly poor target chroma were C♯, D♯ F♯, G♯, and A♯. (TIF) [file pone.0273828.s010.tif]

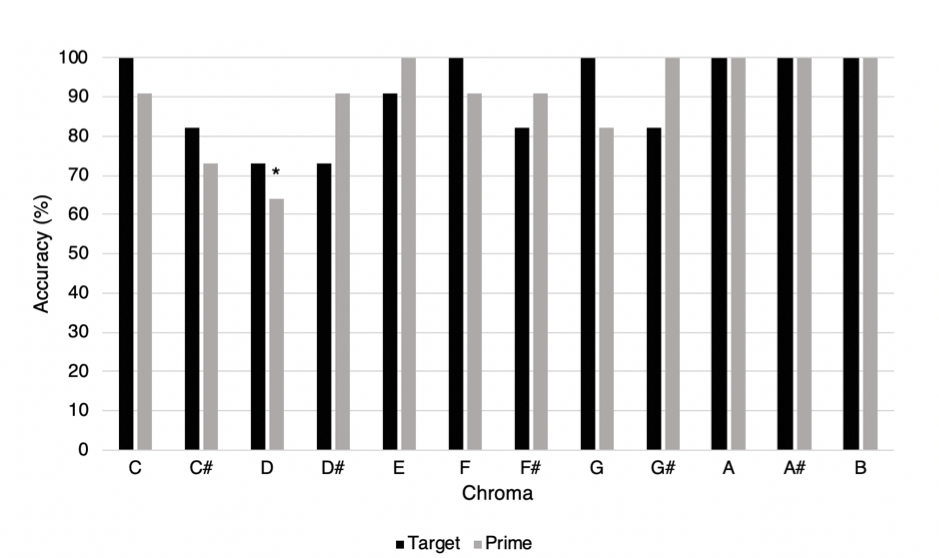

Supplement: S10 Fig — The D prime may have had a disruptive effect for this participant. (TIF) [file pone.0273828.s011.tif]
